# Plant Metabolites Involved in the Differential Development of a Heliantheae-Specialist Insect

**DOI:** 10.3390/metabo11030134

**Published:** 2021-02-25

**Authors:** Marília Elias Gallon, Leonardo Gobbo-Neto

**Affiliations:** Núcleo de Pesquisa em Produtos Naturais e Sintéticos, School of Pharmaceutical Sciences of Ribeirão Preto, University of São Paulo (USP), Av. do Café s/n°, Ribeirão Preto, SP 14040-903, Brazil; mariliagallon@usp.br

**Keywords:** *Tithonia diversifolia*, *Tridax procumbens*, *Aldama robusta*, *Chlosyne lacinia*, LC-MS, GC-MS, chemical ecology, metabolomics

## Abstract

Balanced nutritional intake is essential to ensure that insects undergo adequate larval development and metamorphosis. Integrative multidisciplinary approaches have contributed valuable insights regarding the ecological and evolutionary outcomes of plant–insect interactions. To address the plant metabolites involved in the larval development of a specialist insect, we investigated the development of *Chlosyne lacinia* caterpillars fed on Heliantheae species (*Tithonia diversifolia*, *Tridax procumbens* and *Aldama robusta*) leaves and determined the chemical profile of plants and insects using a metabolomic approach. By means of LC-MS and GC-MS combined analyses, 51 metabolites were putatively identified in Heliantheae species and *C. lacinia* caterpillars and frass; these metabolites included flavonoids, sesquiterpene lactones, monoterpenoids, sesquiterpenoids, diterpenes, triterpenes, oxygenated terpene derivatives, steroids and lipid derivatives. The leading discriminant metabolites were diterpenes, which were detected only in *A. robusta* leaves and insects that were fed on this plant-based diet. Additionally, caterpillars fed on *A. robusta* leaves took longer to complete their development to the adult phase and exhibited a greater diapause rate. Hence, we hypothesized that diterpenes may be involved in the differential larval development. Our findings shed light on the plant metabolites that play roles in insect development and metabolism, opening new research avenues for integrative studies of insect nutritional ecology.

## 1. Introduction

Dietary requirements are a driving force in insect development and are directly related to ecological and evolutionary processes [1,2]. Plant–insect interactions are the most frequent interaction between herbivores and plants. Hence, understanding these interactions is a crucial step in grasping community dynamics [3,4,5]. Recently, ecometabolomics has been described as a powerful approach to comprehend ecologically important phenomena (e.g., interactions between biotic and abiotic factors; complex trophic relationships; animal behavior and global climate changes) [6,7,8]. The integrated knowledge obtained from ecometabolomics studies has been used to provide insights into the insect metabolism depending on the nutritional content of the food consumed during the larval development stage [9].

Insect herbivores acquire the nutrients that are needed for their development, growth and reproduction from plants. Most insects are specialized to feed on selected plant species [10]. However, both specialist and generalist insects experience diverse nutritional landscapes due to the variation in chemical content between and within plant species generated by genetic and environmental factors. In this respect, natural selection favors the insects that are more adapted to regulate their nutrient intakes [11,12,13]. The nutritional content, which is primarily related to the dietary protein and carbohydrate ratios, and availability of the insect larval diet were associated with differential larval development (body mass, diapause rate and mortality), reproductive traits and insect body composition [14,15]. Additionally, a recent study showed that a conifer-specialist insect feeding on closely related plant species exhibited distinct insect metabolomes, which were related to the composition of the plant metabolite that they fed on [9].

*Chlosyne lacinia* (Geyer) (Lepidoptera: Nymphalidae), commonly known as bordered patch or sunflower patch, is a specialist insect herbivore that feeds on a variety of host plants within Asteraceae (especially on Heliantheae species) [16,17]. *C. lacinia* is a holometabolous insect and exhibits a four-stage life cycle (egg, larvae, pupae and adult). The larval stage has five instars, and the complete insect life cycle usually takes 30–40 days [18]. Butterfly wings are usually black with yellow-orange spots, and most of the caterpillars are black with orange stripes and black spines; however, color polymorphism has been described for both, adults and larvae [19,20,21]. In the field, Brazilian populations of *C. lacinia* commonly infest *Tithonia diversifolia* (Hemsl.) A. Gray during the autumn (March to June) [22,23]. In addition, we observed that during that season, *C. lacinia* also used *Tridax procumbens* L. as a host plant and, less frequently, *Aldama robusta* (Gardner) E.E. Schill. and Panero. 

*T. diversifolia*, *T. procumbens* and *A. robusta* are all species that belong to the tribe Heliantheae (Asteraceae family) [24]. Although these species belong to the same tribe, differences in the chemical profile of each species are remarkable: *T. diversifolia* is characterized primarily by the presence of flavonoids and sesquiterpene lactones [25,26,27]; *A. robusta* is characterized by the presence of flavonoids and sesquiterpene lactones plus various diterpenes [28,29]; also, for *T. procumbens,* flavonoids and alkaloids were described but no sesquiterpene lactones or diterpenes [30,31,32]. In addition to the essential function in plant defense against predators, plant metabolites have been associated with the modulation of insect behavior, especially dietary specialization [33,34]. Hence, investigating how a specialist insect herbivore is affected by complex plant metabolites and which metabolites are responsible for the differential insect development has contributed valuable insights on plant–insect interactions and their ecological roles.

In this study, we explored the plant metabolites involved in *C. lacinia* larval development. To this end, we performed insect development assays along with liquid chromatography coupled to mass spectrometry (LC-MS)-based and gas chromatography coupled to mass spectrometry (GC-MS)-based metabolomics of plants and insects to (a) evaluate how *C. lacinia* larval development is affected by different Heliantheae plants, (b) investigate the main discriminant metabolites in the plants and *C. lacinia* according to each diet provided to the caterpillars, and (c) correlate the chemical differences in the diets with the differences in the insect development.

## 2. Results

### 2.1. Metabolomic Analysis

**Figure 1:**
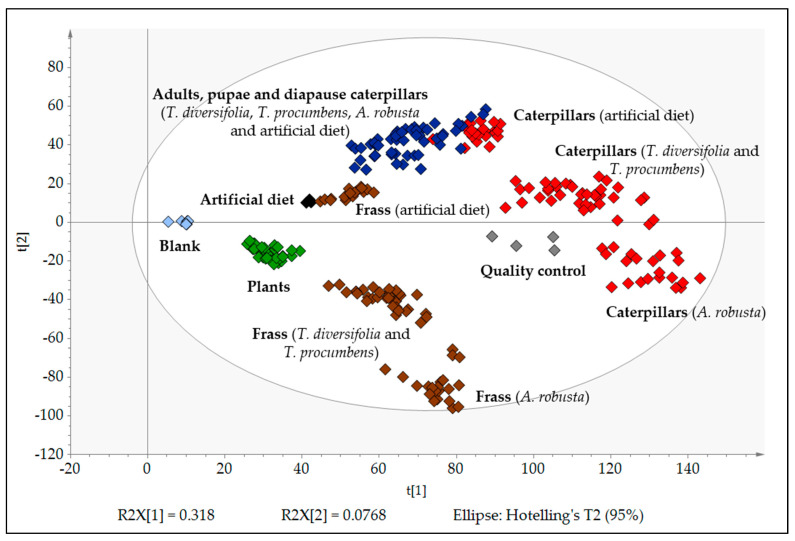
Principal component analysis (PCA) score plot of LC-MS data for diets and *C. lacinia* samples. Samples are colored according to their category: plant samples are represented as green diamonds; artificial diet samples are represented as black diamonds; *C. lacinia* frass samples are represented as brown diamonds; *C. lacinia* caterpillar samples are represented as red diamonds and *C. lacinia* adults, pupae and diapause caterpillar samples are represented as blue diamonds; blank samples are represented as light blue diamonds and quality control samples are represented as gray diamonds (R_2_X = 0.728; Q^2^ = 0.612).

Initially, principal component analysis (PCA) of the diets and insect samples was performed using the LC-MS data. Plant leaves and artificial diet samples, provided as diets to the caterpillars, showed a tendency to cluster apart from *C. lacinia* samples (caterpillars, frass, pupae, diapause caterpillars and adults) (Figure 1). The blank samples (extraction solution) were clustered together, and also the pooled quality control samples (composed of a small aliquot of each sample), which confirmed the reproducibility of the LC-MS analyses and the suitability of the data processing.

*C. lacinia* caterpillars and frass showed a clustering tendency according to the larval diet provided. Caterpillars fed on *T. diversifolia* or *T. procumbens* leaves clustered together and their frass samples also clustered together. Caterpillars fed on *A. robusta* leaves or artificial diet and their respective frass samples formed groups apart from each other. *C. lacinia* adults, pupae and diapause caterpillars exhibited a clustering tendency, which was not related to the diet provided to the caterpillars.

**Figure 2:**
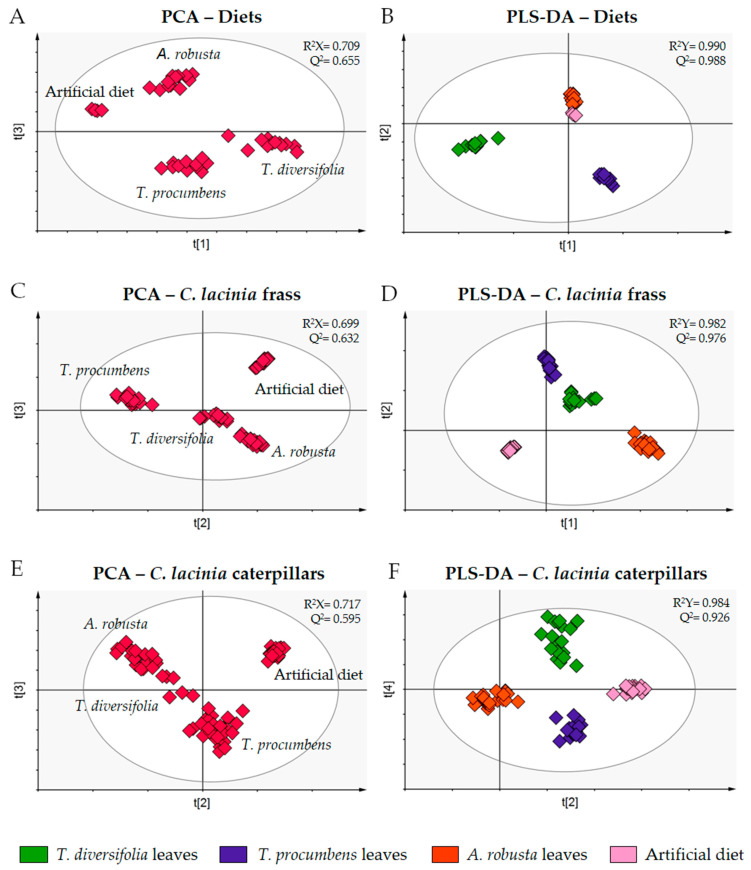
PCA and PLS-DA score plots of the combined LC-MS and GC-MS data obtained for the diets, *C. lacinia* frass and *C. lacinia* caterpillars fed on Heliantheae species leaves or artificial diet (X variables). Classes for PLS-DA (Y variables) were determined according to the diets provided to the caterpillars (green, *T. diversifolia* leaves; purple, *T. procumbens* leaves; orange, *A. robusta* leaves; light pink, artificial diet). (**A**) PCA score plot of the diet samples (Heliantheae species leaves and artificial diet). (**B**) PLS-DA score plot of the diet samples (Heliantheae species leaves and artificial diet). (**C**) PCA score plot of *C. lacinia* frass samples. (**D**) PLS-DA score plot of *C. lacinia* frass samples. (**E**) PCA score plot of *C. lacinia* caterpillar samples. (**F**) PLS-DA score plot of *C. lacinia* caterpillar samples.R^2^X, goodness of fit of the X variables; R^2^Y, goodness of fit of the Y variables; Q^2^, goodness of prediction.

To assess the discriminant metabolites, we combined the LC-MS and GC-MS data to perform individual PCA and partial least squares discriminant analysis (PLS-DA) for selected sets of samples (diets, *C. lacinia* frass and *C. lacinia* caterpillars) (Figure 2).

PCA of the diets showed that they were clustered apart from each other, as expected for samples of different plant species that present distinct chemical content. In the PCA score plot of the *C. lacinia* frass samples, we observed that samples were clustered according to the diet provided to the caterpillars. Additionally, we noticed that *C. lacinia* frass of the caterpillars that were fed on *T. diversifolia* or *T. procumbens* leaves resembled one another, i.e., they were clustered close to one another. PCA of *C. lacinia* caterpillar samples showed a distinction between the caterpillars that were fed on plant-based diets and the caterpillars that were fed on the artificial diet.

PLS-DA score and loading plots along with the variable importance in projection (VIP) plots were employed to determine the discriminant metabolites for each set of samples, i.e., the metabolites that are important for the separation between groups.

We putatively identified 17 compounds that were indicated as discriminant in the LC-MS analysis, including flavonoids, sesquiterpene lactones and diterpenes. Additionally, 34 discriminant compounds were putatively identified in the GC-MS analysis, including monoterpenoids, sesquiterpenoids, diterpenes, triterpenes, oxygenated terpene derivatives, steroids and lipid derivatives (saturated and unsaturated fatty acids) (Table 1). All metabolites were putatively identified at level 2 of confidence [35,36]. The spectroscopic data of the putatively identified compounds are shown in the Supplementary Material (Tables S1 and S2).

For the LC-MS analyses, in general, the metabolites that were detected in the plant leaves were also found in the insect samples (frass and caterpillars) previously fed on each respective based-plant diet. Flavones and furanoheliangolide-type sesquiterpene lactones were found in *T. diversifolia* leaves and in the samples of *C. lacinia* frass and caterpillars that were fed on this diet. When we provided *T. procumbens* leaves as the larval diet, the main discriminant metabolites detected were flavonoids 3-*O*-methylated. For the samples for which we fed the caterpillars on *A. robusta* leaves, we found flavones, a flavonoid 3-*O*-glycosylated, a furanoheliangolide-type sesquiterpene lactone and some diterpenes. In the GC-MS analysis, we also detected some metabolites in all the samples of a respective diet. However, various metabolites (especially lipid derivatives) were detected only in *C. lacinia* caterpillar samples regardless of the diet provided, and other metabolites were detected only in the diets and *C. lacinia* frass samples.

### 2.2. C. lacinia Development

*C. lacinia* caterpillars fed on Heliantheae plant-based diets or the artificial diet were able to complete metamorphosis to the adult phase. However, the development of these caterpillars was affected by the diet that they were fed on. *C. lacinia* caterpillars fed on *T. diversifolia* leaves exhibited larval and pupal viability of 100%, which confirmed their host preference for *T. diversifolia*. In contrast, *C. lacinia* caterpillars fed on *A. robusta* leaves showed a diapause rate much greater (70%) than caterpillars fed on *T. procumbens* (10%) or *T. diversifolia* leaves (2%). However, *C. lacinia* caterpillars fed on *T. procumbens* leaves exhibited a greater mortality rate (25%) than the caterpillars fed on other diets (Table 2).

*C. lacinia* caterpillars that were fed on an artificial diet showed a mortality rate of 10% and were able to complete the metamorphosis. However, these caterpillars exhibited larval and pupal viability lower than caterpillars that were fed on plant-based diets. Thus, Heliantheae species may possess some essential metabolites that were not provided in the artificial diet.

**Figure 3:**
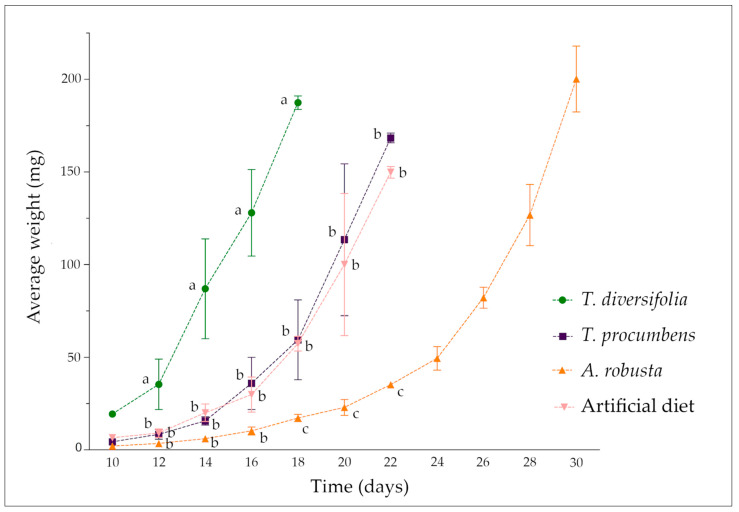
Growth curve for *C. lacinia* caterpillars fed on Heliantheae plant-based diets or artificial diet, based on the development period and the average weight of the caterpillars. Error bars indicate standard errors of the mean (*n* = 3). Different letters indicate statistically significant differences between the diets (plant leaves or artificial diet) within a time point (*p* < 0.05, two-way ANOVA with a Bonferroni post hoc test).

According to the diet the caterpillars were fed on, we found differences in the development period related to the time from egg hatching to the pupal stage. *C. lacinia* caterpillars fed on *T. diversifolia* leaves completed their development to pupae within 18 days. Caterpillars that were fed on *T. procumbens* leaves or artificial diet took approximately 20 days to complete their development to pupae. For *C. lacinia* caterpillars fed on *A. robusta* leaves, the development period was longer and lasted approximately 30 days (Figure 3).

When we provided a *T. diversifolia*-based diet, the 1st larval instar for *C. lacinia* caterpillars took approximately six days. For the other diets, the 1st instar period was longer: caterpillars fed on *T. procumbens* leaves or artificial diet remained at this stage for approximately 12 days, while caterpillars fed on *A. robusta* leaves remained in the 1st larval instar for approximately 15 days. Other instars for the caterpillars fed on *T. diversifolia*, *T. procumbens* or an artificial diet lasted approximately three days. For the caterpillars fed on *A. robusta*, 2nd, 3rd and 4th instars lasted approximately four days. For all the diets, the pupal period lasted approximately eight days, and the adults lived approximately 12 days (Table 3).

*C. lacinia* caterpillars fed on *T. procumbens* leaves exhibited development similar to the development of caterpillars fed on an artificial diet, in relation to the average weight and period of development, i.e., no significant differences were found in corresponding days (*p* > 0.05).

Statistical significance was found for the average weight of the *C. lacinia* caterpillars fed on *T. diversifolia* leaves and the caterpillars fed on other diets, starting on the 12th day of development (*p* < 0.05 for the 12th day and *p* < 0.001 from the 14th to the 18th day). The average weight of *C. lacinia* caterpillars fed on *A. robusta* leaves exhibited statistical significance compared to the average weight of caterpillars fed on *T. procumbens* leaves (*p* < 0.01) or artificial diet (*p* < 0.001) starting on the 18th day of development.

## 3. Discussion

**Figure 4:**
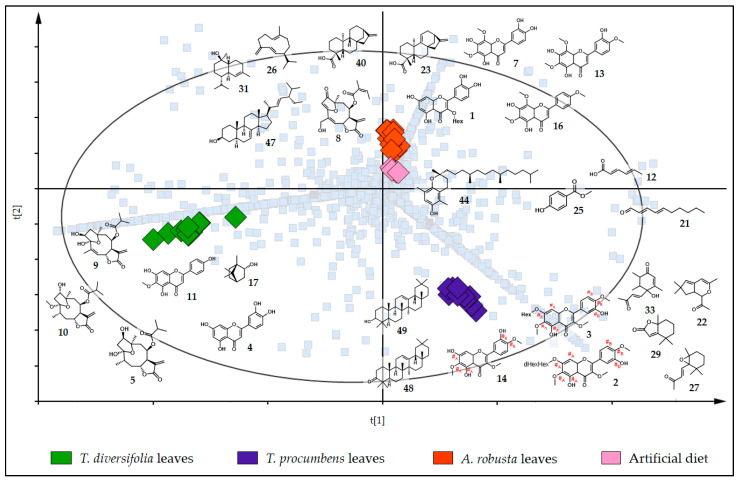
PLS-DA score plot superimposed on the loading plot of the diets (Heliantheae species leaves and artificial diet). Samples of the diets provided to *C. lacinia* caterpillars are represented as diamonds and colored according to the diet (green, *T. diversifolia* leaves; purple, *T. procumbens* leaves; orange, *A. robusta* leaves; light pink, artificial diet). Light blue squares represent the ions detected in the LC-MS or GC-MS analyses. Structures of the discriminant metabolites in each diet are represented. Interchangeable groups in the ring A and B of the flavonoids 3-*O*-methylated are indicated by #_A_ and #_B,_ respectively. (R_2_X[1] = 0.205; R_2_X[2] = 0.158).

We compared the chemical profile of the three plant species provided as diets to *C. lacinia* caterpillars, and we found that flavonoids 3-*O*-methylated (**2**, **3** and **14**) were the major discriminant compounds putatively identified in *T. procumbens* leaves. All these flavonoids presented a hydroxy (OH) group and a methoxy (OCH_3_) group in the B ring. The A ring was characterized by OH, OCH_3_ and *O*-glycoside groups or only by OH and OCH_3_ groups. The exact position of each group in the A and B rings could not be assigned by the techniques employed in this study. Additionally, we putatively identified two triterpenes (**48** and **49**) and four oxygenated terpene derivatives (**22**, **27**, **29** and **33**). The exclusive metabolites detected in *T. diversifolia* leaves included two flavones (**4** and **11**), a monoterpene (**17**) and three furanoheliangolide-type sesquiterpene lactones typically reported for the species (**5**, **9** and **10**). In the leaves of *A. robusta*, we detected three flavones (**7**, **13** and **16**), a flavonoid 3-*O*-hexoside (**1**), a furanoheliangolide-type sesquiterpene lactone (**8**), two diterpenes (**23** and **40**), two sesquiterpenes (**26** and **31**) and a steroid as discriminant compounds (**47**). Interestingly, most of the flavonoids putatively identified are flavones with a fully substituted A ring (two OH groups at positions C-5 and C-7 and two OCH_3_ groups at positions 6 and 8). For the artificial diet samples, some of the constituents added as a nutritional source and food preservative into the artificial diet preparation were, as expected, detected exclusively in these samples and appointed as discriminant compounds (**12**, **21**, **25** and **44**) (Figure 4).

**Figure 5:**
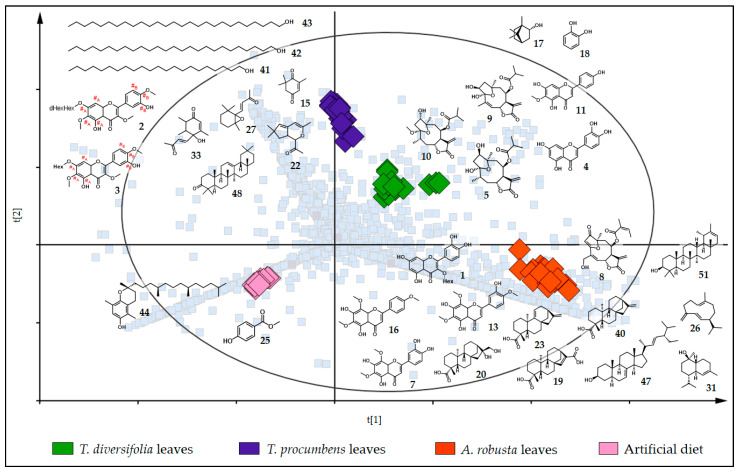
PLS-DA score plot superimposed on the loading plot of *C. lacinia* frass. Samples of *C. lacinia* frass are represented as diamonds and colored according to the diet provided to the caterpillars (green, *T. diversifolia* leaves; purple, *T. procumbens* leaves; orange, *A. robusta* leaves; light pink, artificial diet). Light blue squares represent the ions detected in the LC-MS or GC-MS analyses. Structures of the discriminant metabolites in each set of *C. lacinia* frass are represented. Interchangeable groups in the ring A and B of the flavonoids 3-*O*-methylated are indicated by #_A_ and #_B,_ respectively. (R_2_X[1] = 0.230; R_2_X[2] = 0.148).

Comparing the samples of *C. lacinia* frass for each diet, we found almost the same metabolites that were appointed as discriminants for the diet samples, with subtle differences (Figure 5). Frass of the caterpillars that were fed on *T. diversifolia* leaves exhibited all the discriminant metabolites putatively identified for *T. diversifolia* leaves plus catechol (**18**). For the frass of *C. lacinia* caterpillars fed on *T. procumbens* leaves, we observed as discriminants of three saturated fatty acid alcohols (**41**, **42** and **43**), one cyclohexanone derivative (**15**) and all other metabolites putatively identified in *T. procumbens* leaves, with the exception of one flavonoid (**14**). When we used *A. robusta* leaves as the diet, *C. lacinia* frass was characterized by the presence of all the metabolites previously detected in *A. robusta* leaves plus one triterpene (**51**) and two more diterpenes (**19** and **20**).

The detection of flavonoids and sesquiterpene lactones in *C. lacinia* frass and Heliantheae species leaves is in agreement with the results of our previous study, in which we reported that *C. lacinia* may have developed a mechanism to avoid the absorption of toxic metabolites through its digestive tube, explaining the presence of the intact forms of sesquiterpene lactones in *C. lacinia* frass [37,38].

Catechol (**18**) and a cyclohexanone (**15**) were detected exclusively in the frass of the caterpillars fed on *T. diversifolia* and *T. procumbens* leaves, respectively. These metabolites probably originated from larger aromatic compounds that were degraded into smaller molecules and then excreted in *C. lacinia* frass. Aerobic catabolism of phenolic compounds by microorganisms has been associated with the conversion of phenol and its derivatives to catechol, which is an intermediate in the central pathway for catabolism of aromatic compounds [39,40]. Thus, the presence of catechol in *C. lacinia* frass may be a result of the catabolism of plant phenolic compounds by the insect gut microbiome.

Interestingly, we detected dihydroactinidiolide (**29**) in all frass samples of the caterpillars fed on plant-based diets, regardless of the species provided as diet, and in *T. procumbens* leaves but not in *T. diversifolia* or *A. robusta* leaves. Dihydroactinidiolide has been described as a plant volatile compound and as an insect pheromone, acting as a queen-recognition pheromone of the red fire ant, *Solenopsis invicta* [41,42]. Additionally, dihydroactinidiolide has been reported as an oxidation product of carotenoids. The non-enzymatic oxidative cleavages of carotenoids led to the formation of various aldehydes and ketones, including β-ionone, in which a second oxidation led to the formation of 5,6-epoxy-β-ionone and then dihydroactinidiolide [43,44]. Hence, the presence of dihydroactinidiolide in the frass of the caterpillars fed on *T. diversifolia* or *A. robusta* leaves probably indicated that carotenoids from these plants were degraded in the insect digestive system but not (or to a lesser extent) in their leaves. For *T. procumbens*, the oxidation of carotenoids leading to oxygenated terpene derivatives was more intense and probably has occurred in the plant tissue, and in the insect digestive system, since we found greater variety of these compounds in *T. procumbens* leaves and in the frass of the caterpillars fed on this plant-based diet (**22**, **27**, **29** and **33**).

In the frass of the caterpillars fed on *A. robusta*-based diet, we detected two additional kaurene-type diterpenes that were not detected in *A. robusta* leaves. Compared to kaurenic acid and grandiflorenic acid, found in *A. robusta* leaves, these diterpenes present two more oxygen atoms in their structure. The biotransformation of diterpenes by microorganisms have been described previously and included reactions of hydroxylation, deacylation, carboxylation, epoxidation, oxidation, conjugation with amino acids, epimerization and others [45,46]. Hence, our findings suggested that oxidation is a probable and suitable mechanism for the excretion of diterpenes in *C. lacinia* frass.

Chemically, the most notable difference between the plant species used as *C. lacinia* larval diets was the presence of diterpenes in *A. robusta* leaves and lacking sesquiterpene lactones in *T. procumbens* leaves. Considering that caterpillars fed on the *A. robusta* leaves diet exhibited a longer period of larval development and higher diapause rate, we hypothesized that diterpenes may be involved in differential insect development.

We found that furanoheliangolide-type sesquiterpene lactones were detected in *T. diversifolia* and *A. robusta* leaves, and in *C. lacinia* frass and caterpillars fed on each respective diet. In addition, the sesquiterpenes lactones were not detected in *T. procumbens* leaves, which is in consonance with previous chemical investigations reported for the species [30,31]. Hence, the longer period of larval development observed for the caterpillars fed on *A. robusta* leaves was probably not related to the presence of furanoheliangolide-type sesquiterpene lactones in the diets provided to the caterpillars, since the same type of sesquiterpene lactones was also detected in *T. diversifolia* leaves, which is the preferential host plant for *C. lacinia*. In addition, caterpillars fed on the *T. procumbens* leaves or artificial diet, which did not exhibit those sesquiterpene lactones, showed a larval development more like the development of the caterpillars fed on *T. diversifolia* leaves than the caterpillars fed on *A. robusta* leaves.

We observed that 1st and 2nd instar caterpillars fed mostly on the adaxial surface of *T. diversifolia*, *T. procumbens* and *A. robusta* leaves. Starting from the 3rd instar, the caterpillars consumed the whole leaves. Additionally, caterpillars fed on *A. robusta* leaves consumed a smaller quantity of foliar surface than the caterpillars that were fed on *T. diversifolia* or *T. procumbens* leaves, especially during their initial instars. The differential leaf consumption may be a consequence of *C. lacinia* caterpillars trying to regulate their nutritional intake, which is in keeping with the integration of the optimal foraging theory and insect nutritional ecology [47].

Comparing the flavonoids that were putatively identified in the leaves of the Heliantheae species, we observed a particular distribution related to the substituents in the A ring and in the C-3 position for each species: *T. diversifolia* leaves exhibited flavones with OH groups at positions C-5 and C-7; in *T. procumbens* leaves, we detected flavonoids 3-*O*-methylated with OH, OCH_3_ and O-glycoside groups in the ring A; and *A. robusta* leaves were characterized by the presence of flavones with a fully substituted A ring (two OH groups and two OCH_3_ groups). The substitution patterns of flavonoids may be associated with *C. lacinia* feeding behavior and host–plant specialization [48,49]. However, because these metabolites were detected in the plant-based diets provided to the caterpillars and in *C. lacinia* frass of the respective diet, it is not clear if the flavonoids can be absorbed in the insect gut and play some roles in the insect physiological functions or if there are physical barriers in the insect digestive tube that prevents the flavonoids from being absorbed [50].

Other remarkable differences between *A. robusta* and the other diets included the lack of campesterol detected in *A. robusta* leaves, and in the frass and caterpillars fed on this diet, while it was detected in all other diets and *C. lacinia* samples. However, we found chondrillasterol exclusively in *A. robusta* and *C. lacinia* frass and caterpillars fed on its leaves but not in all other diets and *C. lacinia* samples. Since insects are incapable of biosynthesizing steroids de novo, a dietary source of steroids is required to ensure proper development and reproduction. For many insect species, campesterol is a precursor of the molting hormones (especially ecdysteroids) [51,52]. Hence, campesterol and chondrillasterol may be involved in regulating molting and metamorphosis in *C. lacinia*, since we observed a higher diapause rate for the caterpillars fed on *A. robusta* plant-based diet.

Oleanane- and ursane-type triterpenes were detected mainly in *T. procumbens* leaves and *C. lacinia* samples fed on this diet. We detected β-amyrone (**48**) exclusively in *T. procumbens* leaves and *C. lacinia* frass and caterpillars fed on this plant-based diet. Additionally, we putatively identified β-amyrin (**49**) and α-amyrone (**50**) in the *T. procumbens* leaves and artificial diet, and in their corresponding *C. lacinia* samples; however, these compounds were not detected in *T. diversifolia* and *A. robusta* leaves. Some triterpenes were described as having insect antifeeding properties and plant defense functions [53]. Hence, considering that the caterpillars fed on *T. procumbens* leaves or artificial diet exhibited a similar development in terms of larval average weight and time to reach the adult phase, triterpenoids may play a role in *C. lacinia* development.

Additionally, differences in the larval and pupal viabilities can be related to the presence of lipid derivatives in the plant-based diets. We putatively identified two acyclic diterpenes (**34** and **38**), a long-chain aldehyde (**35**) and a polyunsaturated fatty acid (**39**) in Heliantheae species and frass of the caterpillars fed on plant-based diets. However, these compounds were not detected in the artificial diet or frass of caterpillars fed on this artificial diet. Since caterpillars fed on the artificial diet exhibited lower larval and pupal viabilities than caterpillars fed on plant-based diets, we may suggest that these metabolites are required for *C. lacinia* metamorphosis. Additionally, saturated (**36**, **41**, **42** and **43**) and polyunsaturated (**37**) fatty acids were detected in the caterpillars of *C. lacinia* that were fed on plant-based diets, which suggested that they are plant derived metabolites that constitute the insect cellular membranes or are stored in the insect fat body [54,55,56]. Myristic acid (**32**) was detected in the Heliantheae species, and in *C. lacinia* frass and caterpillars fed on these plant-based diets. Interestingly, we found this compound in the caterpillars fed on artificial diet but not in the artificial diet or the frass of caterpillars fed on this diet. Several fatty acid derivatives (e.g., stearic, palmitic, myristic and lauric acids) are regulated for pheromone biosynthesis and used as precursor molecules [57,58]. Accordingly, the presence of myristic acid in the caterpillars fed on artificial diet indicated the ability of *C. lacinia* caterpillars to biosynthesize a probably important saturated fatty acid, which was not provided into the artificial diet.

Our study provided insights into the metabolites involved in the interaction between Heliantheae plants and a specialist insect herbivore. By correlating how the larval development of *C. lacinia* was influenced by the Heliantheae species they were fed on, we investigated the discriminant metabolites in each diet that may be responsible for such differential development. For this Heliantheae-specialist insect, the sesquiterpene lactones typically present in its host plants were not related to detrimental effects in larval development. Diterpenes may be associated with longer development periods and modulation of feeding behavior in *C. lacinia* caterpillars, and triterpenes, steroids and lipid derivatives, which are related to larval and pupal viabilities. Taken together, these findings highlighted plant metabolites that played a role in insect development and behavior, pointing out relevant directions for understanding the interaction of semiochemicals with biological controls used in integrated pest management programs and for integrative studies in insect physiology, behavior and nutritional ecology.

## 4. Materials and Methods

### 4.1. Experimental Design

*C. lacinia* caterpillars fed on Heliantheae species leaves and artificial diet were reared under laboratory conditions (14 h photoperiod, temperature of 25 ± 2 °C, 50% ± 10% relative humidity) using rearing boxes (size 30 cm × 25 cm × 20 cm, plastic floor and sides, nylon mesh ceiling). Fresh plant leaves and artificial diet were provided daily until the pupal stage. Butterflies were fed honey:water solution (1:10, *v*:*v*). *C. lacinia* development was measured by means of larval average weight, larval mortality, diapause rate, larval viability and pupal viability. For the metabolomic analysis, we sampled *C. lacinia* caterpillars, frass, pupae and adults during the development experiment, and the leaves of the plants provided as the diet and the artificial diet.

### 4.2. Insects and Diets

*C. lacinia* egg masses were collected in the field (nearby the Garden of Medicinal Plants of the School of Pharmaceutical Sciences of Ribeirão Preto, University of São Paulo, Ribeirão Preto, SP, Brazil) and identified according to their characteristic yellow color and host preference for Heliantheae species. Insects were reared and maintained at laboratory conditions (fed on their preferential host plant—*T. diversifolia*; 14 h photoperiod, temperature of ±2 °C and 50 ± 10% relative humidity) for further experiments.

During the larval development experiments, fresh leaves of *T. diversifolia*, *T. procumbens* and *A. robusta* were collected at the Garden of Medicinal Plants of the School of Pharmaceutical Sciences of Ribeirão Preto, University of São Paulo, Ribeirão Preto, SP, Brazil and provided to the caterpillars as diets. In the meantime, we sampled some of the plant leaves for metabolomic analysis, which were immediately frozen in liquid nitrogen and stored in a −20 °C freezer.

Additionally, an artificial diet was used as control. The artificial diet was composed of bean (240 g), wheat germ (120 g), brewer’s yeast (72 g), sorbic acid (2.4 g), ascorbic acid (7.3 g), nipagin (4.4 g), caragenate (20 g), methanal (6 mL), vitamin solution (10 mL) and water (1000 mL) [59,60].

### 4.3. C. lacinia Development

*C. lacinia* egg masses were separately placed over the leaves of each Heliantheae species or artificial diet and maintained in rearing boxes. On the 10th day after egg hatching, 20 caterpillars were randomly selected and placed in other rearing boxes with the corresponding diet. Larval weight of each caterpillar was measured every two days using an analytical balance. Assays were performed in triplicate (*n* = 3). Statistical significance was evaluated by means of two-way ANOVA with a Bonferroni post hoc test using the software GraphPad Prism (version 5.02 for Windows, GraphPad Software, San Diego, CA, USA). Mortality rate was measured in terms of percentage of caterpillars that died during *C. lacinia* larval development. Additionally, we evaluated the diapause rate, larval viability (percentage of caterpillars that completed the transformation to pupae) and pupal viability (percentage of pupae that completed metamorphosis to adults) for each diet.

The remaining *C. lacinia* caterpillars (ca. 30 to 50 caterpillars), kept in the first rearing boxes and fed on each diet, were sampled during their development for further LC-MS and GC-MS analyses. We sampled four *C. lacinia* caterpillars in each stage of development (2nd, 3rd, 4th and 5th instars), frass of caterpillars in each corresponding larval instar, four pupae and four adults (two female and two male) for each diet (*n* = 3). Each sample was immediately frozen and stored in a −20 °C freezer.

### 4.4. Metabolomic Analysis

#### 4.4.1. Sample Preparation

Plant samples (leaves of *T. diversifolia*, *T. procumbens* and *A. robusta*), artificial diet samples (sampled in four different days during the larval development experiment) and insect samples (*C. lacinia* caterpillars, frass, pupae, diapause caterpillars and adults) were freeze-dried (*Labconco*, 48 h, −30 °C) and pulverized. All samples were maintained in a −20 °C freezer until LC-MS and GC-MS analyses.

#### 4.4.2. LC-MS Analyses

Extractions were performed using 5 mg of each sample and 500 µL of MeOH:H_2_O solution (7:3, *v*:*v*) with hydrocortisone (10 µg·mL^−1^), used as an internal standard. Each sample added with the extraction solution was submitted to vortex agitation (AV-2, *Gehaka*, 1 min, room temperature) and an ultrasonic bath (*UltraSonic Cleaner* 1400, 40 kHz, UNIQUE, 10 min, room temperature). After centrifugation (M-240R, *BOECO Germany*, 5 min, 10 °C), the extracts were filtered through a 0.20-µm PTFE membrane into HPLC vials.

Chemical profiles of *C. lacinia* samples and plants were obtained on an *Accela* UHPLC (Ultra-High-Performance Liquid Chromatography) system (Thermo Scientific™, Waltham, MA, USA) coupled to a diode array ultraviolet light detector and to an Orbitrap mass spectrometer (Exactive^TM^ Plus, Thermo Scientific™, Waltham, MA, USA). Chromatograms were acquired simultaneously in both the positive and negative mode, using a C18 Kinetex column (2.6 µm, Polar C18, 150 mm × 2.1 mm, Phenomenex) and mobile phase composed of water (A) and acetonitrile (B) both with 0.1% of formic acid (0–2 min, 10% B; 2–30 min, 10–100% B; 30–34 min, 100% B; 34–37 min, 100–10% B and 37–40 min, 10% B) with a flow rate of 0.7 mL·min^−1^. Oven temperature was set at 45 °C and 5 µL of each sample was injected. 

The mass spectrometer parameters were as follows: full MS (100–1000 *m/z*); full MS-MS (80–1000 *m/z)*; resolution of 70,000 (MS mode) and 35,000 (AIF mode); maximum injection time, 200 ms; sheath gas flow, 30 Ua; auxiliary gas flow, 10 Ua; capillary temperature, 300 °C; spray voltage, 3.6 kV (positive mode) and 3.2 kV (negative mode); maximum spray current, 100 μA; S-lens RF level, 55; drying, nebulizer and fragmentation gas, N_2_. The mass spectra were visualized using the software Xcalibur (Thermo Scientific™, Waltham, MA, USA).

Every 50 analyzed samples, blank samples (extraction solution) and quality control samples (composed of 20 µL of each previously prepared extract) were injected and analyzed using the same UHPLC-MS conditions. Quality control samples were used to assess the reproducibility of the data throughout the sample preparation, data acquisition and data processing, and ensure that the analytical process was performed accordingly. Total ion chromatograms of the quality control samples obtained during data acquisition are presented at the Supplementary Material (Figures S1 and S2).

#### 4.4.3. GC-MS Analyses

Extracts were prepared using 10 mg of each sample and 500 µL of dichloromethane (CH_2_Cl_2_), followed by vortex agitation (5 min, room temperature) and ultrasonic bath (15 min, room temperature). The extracts were filtered through cotton wool using a glass syringe. After solvent evaporation, each sample was solubilized with CH_2_Cl_2_ in a concentration of 10 mg·mL^−1^.

GC-MS analyses were performed in a gas chromatograph coupled to a quadrupole mass spectrometer (QP2010 Ultra, Shimadzu Corporation, Kyoto, Japan) using a ZB-5MS column (30 m × 0.25 mm × 0.25 µm) and a temperature program of 60–300 °C at 5 °C·min^−1^ (60 °C, 3 min; 60–300 °C, 51 min and 300 °C, 71 min). The following conditions were employed: carrier gas, He; column oven temperature, 60 °C; injection temperature, 270 °C; injection mode, split; injection volume, 1.0 µL; flow control mode, linear velocity; pressure, 86.7 kPa; total flow, 11.4 mL min^−1^; column flow, 1.40 mL min^−1^; linear velocity, 43.2 cm s^−1^; purge flow, 3.0 mL·min^−1^ and split ratio, 5. The mass spectra were acquired in the scan mode between 35 and 500 *m/z*, with an ion source temperature of 250 °C and EI voltage of 70 eV. Chromatograms and mass spectra were visualized using the GC Solutions (version 4.20 for Windows, Shimadzu Corporation, Kyoto, Japan).

#### 4.4.4. Data Processing

LC-MS data from positive and negative ionization modes were separately converted to *.mzXML using the software ProteoWizard-MSconvert (version 3 for Windows, Proteowizard Software Foundation, Palo Alto, CA, USA).

Each data set was processed using the software MzMine^TM^ (version 2.51 for Windows, BMC Bioinformatics, United Kingdom) and employing the followed parameters: mass detection, mass detector—exact mass (noise level, 1.0 × 10^4^); algorithm—wavelets (ADAP) chromatogram builder (min group size in # of scans, 3; group intensity threshold, 1.0 × 10^4^; min highest intensity, 1.0 × 10^7^; *m/z* tolerance, 0.001 *m/z* or 5 ppm); chromatogram deconvolution, algorithm—wavelets (ADAP) (S/N threshold, 10; S/N estimator, intensity window SN; min feature height, 1.0 × 10^6^; coefficient/area threshold, 100; peak duration range, 0.03–2.0; RT wavelet range, 0.01–0.10), *m/z* center calculation—median; isotopic peak grouper (*m/z* tolerance, 0.002 *m/z* or 5 ppm; retention time tolerance, 0.2 min (absolute); maximum charge, 2 and representative isotope, most intense) and alignment, join aligner (*m/z* tolerance, 0.005 *m*/*z* or 10 ppm; weight for *m/z*, 50; retention time tolerance, 0.2 min (absolute) and weight for retention time, 50). After processing, data were exported as a *.csv spreadsheet. Data spreadsheets from the positive and negative ionization modes were joined in a single spreadsheet and log-transformed. 

GC-MS data, previously converted to *.mzXML with the GS Solutions software, were processed using the software MzMine^TM^ (version 2.51 for Windows, BMC Bioinformatics, United Kingdom): mass detection, mass detector—centroid (noise level, 1.0 × 10^3^); ADAP chromatogram builder (min group size in # of scans, 3; group intensity threshold, 1.0 × 10^3^; min highest intensity, 1.0E3; *m/z* tolerance, 0.5 *m/z* or 0 ppm); chromatogram deconvolution, algorithm—wavelets (ADAP) (S/N threshold, 10; S/N estimator, intensity window SN; min feature height, 1.0 × 10^3^; coefficient/area threshold, 100; peak duration range, 0.02–2.0; RT wavelet range, 0.01–0.20), *m/z* center calculation—median; hierarchical clustering (min cluster distance (min), 0.01; min cluster size, 3; min cluster intensity, 3.0 × 10^3^; min edge-to-height ratio, 0.3; min delta-to-height ratio, 0.2; min sharpness, 100; choice of model peak based on, *m/z* value); alignment, ADAP aligner (GC) (min confidence, 0.01; retention time tolerance, 0.3 min (absolute); *m/z* tolerance, 0.5 *m/z* or 0 ppm; score threshold, 0.7 and retention time similarity, retention time difference, 0.4. After processing, data were exported as a *.csv spreadsheet and log-transformed.

#### 4.4.5. Multivariate Statistical Analyses

Data mining was performed with the software SIMCA (version 13.0.3.0 for Windows, Umetrics, Umeå, Sweden). Initially, we performed PCA using only the LC-MS data for all collected samples. Then, PCA was performed for each group of similar samples (diets, *C. lacinia* frass and *C. lacinia* caterpillars) combining the data from LC-MS and GC-MS. For the supervised statistical analysis, we employed PLS-DA for each set of samples using the LC-MS and GC-MS data. Classes for the PLS-DA were determined according to the diet provided to the caterpillars during their development (*T. diversifolia* leaves, *T. procumbens* leaves and *A. robusta* leaves or artificial diet). The discriminant metabolites for each group were determined using the PLS-DA loading plot and VIP plot, considering that the variables with VIP value greater than 1 were important for the separation between groups.

Metabolites detected by LC-MS were putatively identified using an in-house database built with the metabolites reported in the literature for *T. diversifolia*, *T. procumbens* and *A. robusta* and comparing their MS and UV spectra, as well as their fragmentation pattern. The stereochemistry of the sugar moieties was not considered for their characterization. Metabolites detected by GC-MS were identified by matching their MS spectra and retention index (RI) with compounds from external database (National Institute of Standards and Technology—NIST; Flavors and Fragrances of Natural and Synthetic Compounds—FFNSC and Wiley libraries). 

## Figures and Tables

**Table 1:** Putatively identified metabolites that were indicated as discriminants in the diets, *C. lacinia* frass and *C. lacinia* caterpillars.

ID	Rt	Mode	Compound Name	*T. diversifolia*			*T. procumbens*			*A. robusta*			Artificial Diet		
				D	F	C	D	F	C	D	F	C	D	F	C
1	5.7	LC	quercetin 3-*O*-hexoside												
2	7.7	LC	flavonoid 3-*O*-methyl												
3	7.9	LC	flavonoid 3-*O*-methyl												
4	8.2	LC	luteolin												
5	8.4	LC	tagitinin A												
6	8.5	LC	nepetin												
7	8.6	LC	5,7,3’,4’-tetrahydroxy 6,8-dimethoxyflavone												
8	8.7	LC	budlein A												
9	8.7	LC	tagitinin B												
10	9.6	LC	1-hydroxy-3-*O*-methyltirotudin												
11	9.8	LC	hispidulin												
12	10.0	GC	2,4-hexadienoic acid												
13	10.2	LC	acerosin												
14	10.8	LC	flavonoid 3-*O*-methyl												
15	11.9	GC	2,6,6-trimethyl-2-cyclohexene-1,4-dione												
16	12.6	LC	nevadensin												
17	12.6	GC	borneol												
18	13.2	GC	catechol												
19	13.4	LC	kaur-15-ene 17,18 dioic acid												
20	13.9	LC	16,17-dihydroxy-ent-kauran-19-oic acid												
21	16.8	GC	2,4-decadienal (*E*,*E*)												
22	18.9	GC	ethanone,1-(1,6,7,7a-tetrahydro- 3,6,6-trimethylcyclopenta pyran-1-yl)												
23	19.9	LC	grandiflorenic acid												
24	20.2	GC	ethanone,1,1’-(1,4-phenylene) bis												
25	20.4	GC	benzoic acid, 4-hydroxy-methyl ester												
26	21.0	GC	germacrene D												
27	21.0	GC	5,6-β-ionone epoxide												
28	21.4	GC	bicyclogermacrene												
29	22.2	GC	dihydroactinidiolide												
30	23.4	GC	spathulenol												
31	25.2	GC	α-cadinol												
32	27.4	GC	myristic acid												
33	28.0	GC	2-cyclohexen-1-one, 4-hydroxy- 3,5,6-trimethyl-4-(3-oxo-1-butenyl)												
34	29.1	GC	neophytadiene												
35	32.7	GC	octadecanal												
36	33.5	GC	heptadecanoic acid												
37	34.1	GC	9,12,15-octadecatrienoic acid, methyl ester												
38	34.3	GC	phytol												
39	34.8	GC	9,12,15-octadecatrienoic acid												
40	39.6	GC	kaurenoic acid												
41	40.6	GC	1-docosanol												
8	45.8	GC	budlein A												
42	46.5	GC	1-hexacosanol												
43	47.8	GC	1-heptacosanol												
44	48.4	GC	β-tocopherol												
45	50.2	GC	lathosterol												
46	50.8	GC	campesterol												
47	51.9	GC	chondrillasterol												
48	52.1	GC	β-amyrone												
49	52.5	GC	β-amyrin												
50	52.8	GC	α-amyrone												
51	54.7	GC	pseudotaraxasterol												

ID, peak identification; Rt, retention time in minutes; D, diet; F, *C. lacinia* frass, C, *C. lacinia* caterpillars. Colors represent diet and *C. lacinia* (frass and caterpillar) samples according to the diet that was provided (green, *T. diversifolia* leaves; purple, *T. procumbens* leaves; orange, *A. robusta* leaves; pink, artificial diet). Darker colors indicate the compounds that were detected in each sample.

**Table 2:** Parameters used to evaluate the development of *C. lacinia* caterpillars fed on Heliantheae species leaves or artificial diet.

Diet	Mortality Rate (%)	Diapause Rate (%)	Larval Viability (%) ^a^	Pupal Viability (%) ^b^
*T. diversifolia* (1)	0	0	100	100
*T. diversifolia* (2)	0	0	100	100
*T. diversifolia* (3)	0	5	95	100
*T. procumbens* (1)	25	0	75	100
*T. procumbens* (2)	30	10	60	100
*T. procumbens* (3)	5	15	80	100
*A. robusta* (1)	0	60	40	100
*A. robusta* (2)	0	75	25	100
*A. robusta* (3)	0	75	25	100
Artificial diet (1)	0	0	85	71
Artificial diet (2)	0	10	90	94
Artificial diet (3)	10	0	90	89

^a^ Percentage of caterpillars that completed the transformation to pupae; ^b^ percentage of pupae that completed metamorphosis to adults; numbers in parentheses represent the replicates.

**Table 3:** Duration of the insect life stages and weight ranges for each larval instar of *C. lacinia* caterpillars fed on Heliantheae plant-based diets or an artificial diet.

Insect Stage	Caterpillars					Pupae	Adults
	1st Instar	2nd Instar	3rd Instar	4th Instar	5th Instar		
Weight (mg)	1 to 10	10 to 20	20 to 50	50 to 120	120 to 200		
Duration (days)							
*T. diversifolia*	5.85 ± 0.67	3.70 ± 0.66	3.25 ± 0.44	3.30 ± 0.66	3.35 ± 0.49	7.55 ± 0.60	11.35 ± 1.04
*T. procumbens*	12.25 ± 0.79	2.70 ± 0.66	3.30 ± 0.47	3.20 ± 0.62	3.05 ± 0.51	7.45 ± 0.60	11.70 ± 0.73
*A. robusta*	15.20 ± 0.41	4.10 ± 0.55	4.15 ± 0.59	4.10 ± 0.64	2.65 ± 0.67	7.70 ± 0.73	11.75 ± 0.85
Artificial diet	12.10 ± 0.72	2.60 ± 0.68	3.35 ± 0.49	3.25 ± 0.44	2.65 ± 0.49	7.90 ± 0.55	11.65 ± 0.99

## Data Availability

The data presented in this study, including the raw mass spectrometric data, are deposited in MetaboLights with the identifier MTBLS2383 at www.ebi.ac.uk/metabolights/MTBLS2383.

## Supplementary Materials

The following are available online at https://www.mdpi.com/2218-1989/11/3/134/s1, Table S1: Spectroscopic data of the putatively identified compounds by UHPLC-UV(DAD)-HRMS(Orbitrap), Table S2: Similarity and retention index of the putatively identified compounds by GC-MS, Figure S1: Total ion chromatograms (TIC) of the quality control samples analyzed by UHPLC-UV-MS during the data acquisition in the negative ionization mode, Figure S2: Total ion chromatograms (TIC) of the quality control samples analyzed by UHPLC-UV-MS during the data acquisition in the positive ionization mode.

## Additional Information

The plants and insects analyzed in this study were registered in the SisGen website (A0AD857) for accessing the Brazilian genetic heritage in line with the current legislation (Law 13,123/15 and Decree 8772/16).
