# Supplementary material for: Plant Metabolites Involved in the Differential Development of a Heliantheae-Specialist Insect
Source: Metabolites. 2021 Feb 25;11(3):134. doi: 10.3390/metabo11030134 (PMC7996590; doi:10.3390/metabo11030134)
Supplement: Supplementary file 1 [file metabolites-11-00134-s001.pdf]

Plant metabolites involved in the differential development of a Heliantheae-specialist insect

Marília Elias Gallon[1], **and Leonardo Gobbo-Neto**[1,*]

[1] Núcleo de Pesquisa em Produtos Naturais e Sintéticos, School of Pharmaceutical Sciences of Ribeirão Preto, University of São Paulo (USP), Av. do Café s/n°, 14040-903, Ribeirão Preto, SP, Brazil.

***** corresponding author: gobbo@fcfrp.usp.br

Table S1: Spectroscopic data of the putatively identified compounds by UHPLC-UV(DAD)-HRMS(Orbitrap).

ID	Rt	Compound name	Molecular Formula (MW)	Positive Ionization Mode TIC (*m/z*)	Positive Ionization Mode AIF (*m/z*)	Negative Ionization Mode TIC (*m/z*)	Negative Ionization Mode AIF (*m/z*)	UV_{max}
1	5.7	quercetin 3-*O*-hexoside	$C_{21}H_{18}O_{13}$ (478.0747)	$[M+H]^+$ 479.0814	303.05	$[M-H]^-$ 477.0665	301.03	255; 265 sh; 297 sh; 352
2	7.7	flavonoid 3-*O*-methyl	$C_{30}H_{36}O_{17}$ (668.1952)	$[M+H]^+$ 669.2018	361.09 bp; 346.07	$[M-H]^-$ 667.1876	359.08 bp; 344.05; 329.07; 314.04; 299.02; 281.07	255; 270 sh; 350
3	7.9	flavonoid 3-*O*-methyl	$C_{24}H_{26}O_{13}$ (522.1373)	$M+H]^+$ 523.1444	361.09 bp; 346.07; 328.06; 331.04	$[M-H]^-$ 521.1290	359.08 bp; 344.05; 329.03	256; 270 sh; 349
4	8.2	luteolin	$C_{15}H_{10}O_6$ (286.0477)	$[M+H]^+$ 287.0547	269.04; 259.09; 161.09; 153.02 bp	$[M-H]^-$ 285.0403	285.04 bp; 267.03; 257.05; 243.03; 241.05; 217.05; 199.04; 175.04; 151.00	257; 266 sh; 298 sh 345

ID	Rt	Compound name	Molecular Formula (MW)	Positive Ionization Mode		Negative Ionization Mode		UV$_{max}$
				TIC (*m/z*)	AIF (*m/z*)	TIC (*m/z*)	AIF (*m/z*)	
5	8.4	tagitinin A	$C_{19}H_{28}O_7$ (368.1835)	$[M+H]^+$ 369.1902	351.18; 333.17; 281.14; 263.13; 245.12; 235.17 bp; 227.11; 217.12	$[M-H]^-$ 367.1758	279.12; 261.11; 243.90 bp	
6	8.5	nepetin	$C_{16}H_{12}O_7$ (316.0583)	$[M+H]^+$ 317.0652	302.04	$[M-H]^-$ 315.0510	300.03	259; 268sh; 341
7	8.6	5,7,3',4'-tetrahydroxy 6,8-dimethoxyflavone	$C_{17}H_{14}O_8$ (346.0689)	$[M+H]^+$ 347.0757	317.06 bp; 302.04; 289.03	$[M-H]^-$ 345.0612	330.04; 315.01; 300.03 bp; 287.02; 271.09	279; 332
8	8.7	budlein A	$C_{20}H_{22}O_7$ (374.1365)	$[M+H]^+$ 375.1432	293.12; 275.09; 257.08; 229.08 bp; 211.07	$[M-H]^-$ 373.1289		267
9	8.7	tagitinin B	$C_{19}H_{26}O_7$ (366.1678)	$[M+H]^+$ 367.1744	349.16; 331.15; 279.16 bp; 261.11; 243.10	$[M-H]^-$ 365.1602	277.11; 259.09; 241.90 bp	

ID	Rt	Compound name	Molecular Formula (MW)	Positive Ionization Mode		Negative Ionization Mode		UV_{max}
				TIC (*m/z*)	AIF (*m/z*)	TIC (*m/z*)	AIF (*m/z*)	
10	9.6	1-hydroxy-3-*O*-methyltirotudin	$C_{21}H_{20}O_{11}$ (448.1006)	$[M+H]^+$ 383.2060	365.10; 353.07 bp; 347.19; 335.06; 261.11; 243.10; 225.09			
11	9.8	hispidulin	$C_{16}H_{12}O_6$ (300.0634)	$[M+H]^+$ 301.0704	286.05	$[M-H]^-$ 299.0561	284.03	269; 334
13	10.2	acerosin	$C_{18}H_{16}O_8$ (360.0845)	$[M+H]^+$ 361.0914	346.07; 331.04 bp; 328.06; 316.02; 303.05	$[M-H]^-$ 359.0771	344.05; 329.03 bp; 314.01; 301.03	279; 342
14	10.8	flavonoid 3-*O*-methyl	$C_{18}H_{16}O_8$ (360.0845)	$[M+H]^+$ 361.0914	346.06; 345.06; 331.04; 328.06; 303.05 bp; 313.03	$[M-H]^-$ 359.0769	344.05; 329.03; 301.03; 286.01 bp	257; 270 sh; 345
16	12.6	nevadensin	$C_{18}H_{16}O_7$ (344.0896)	$[M+H]^+$ 345.0964	330.07; 315.05 bp; 300.06; 287.05; 201.00; 182.99	$[M-H]^-$ 343.0819	328.06; 313.04 bp; 298.01; 285.04	278; 332

ID	Rt	Compound name	Molecular Formula (MW)	Positive Ionization Mode		Negative Ionization Mode		UV_{max}
				TIC (*m/z*)	AIF (*m/z*)	TIC (*m/z*)	AIF (*m/z*)	
19	13.4	kaur-15-ene 17,18 dioic acid	$C_{20}H_{28}O_4$ (332.1987)	$[M+H]^+$ 333.2057	287.20; 241.19 bp	$[M-H]^-$ 331.1916		
20	13.9	16,17-dihydroxy-ent-kauran-19-oic acid	$C_{20}H_{32}O_4$ (336.2300)	$[M+H]^+$ 337.2370	319.23; 301.22; 255.21 bp	$[M-H]^-$ 335.2229		
23	19.9	grandiflorenic acid	$C_{20}H_{28}O_2$ (300.2089)	$[M+H]^+$ 301.2155	255.21; 213.16; 199.15 bp			

ID, peak identification; Rt, retention time in minutes; MW, monoisotopic weight; TIC, total ion chromatogram; AIF, all ion fragmentation; *m/z*, mass to charge ration; UV_{max}, maximum absorption in the ultraviolet spectra in nanometers.

Table S2: Similarity and retention index of the putatively identified compounds by GC-MS.

ID	Rt	Compound name	S	RI_{calc}	RI_{ref}
12	10.0	2,4-hexadienoic acid	96	1083	-
15	11.9	2,6,6-trimethyl-2-cyclohexene-1,4-dione	91	1147	1142
17	12.6	borneol	87	1170	1171
18	13.2	catechol	96	1190	1197
21	16.8	2,4-decadienal (E,E)	90	1318	1317
22	18.9	ethanone, 1-(1,6,7,7a-tetrahydro-3,6,6-trimethylcyclopenta pyran-1-yl)	88	1396	-
24	20.2	ethanone,1,1'-(1,4-phenylene) bis	94	1448	-
25	20.4	benzoic acid, 4-hydroxy-methyl ester	98	1459	1452
26	21.0	germacrene D	96	1480	1479
27	21.0	5,6-β-ionone epoxide	88	1480	1488
28	21.4	bicyclogermacrene	94	1496	1497
29	22.2	dihydroactinidiolide	87	1529	1525
30	23.4	spathulenol	88	1579	1573
31	25.2	α-cadinol	85	1656	1656
32	27.4	myristic acid	94	1757	1767
33	28.0	2-cyclohexen-1-one, 4-hydroxy-3,5,6-trimethyl-4-(3-oxo-1-butenyl)	86	1785	-
34	29.1	neophytadiene	95	1838	1840
35	32.7	octadecanal	92	2021	2021
36	33.5	heptadecanoic acid	91	2063	2080

37	34.1	9,12,15-octadecatrienoic acid, methyl ester	95	2095	2099
38	34.3	phytol	97	2106	2111
39	34.8	9,12,15-octadecatrienoic acid	95	2133	2125
40	39.6	kaurenoic acid	89	2412	-
41	40.6	1-docosanol	94	2475	2456
8	45.8	budlein A	92	2829	-
42	46.5	1-hexacosanol	91	2879	2852
43	47.8	1-heptacosanol	91	2977	2948
44	48.4	β-tocopherol	89	3023	3043
45	50.2	lathosterol	91	3167	3170
46	50.8	campesterol	85	3215	-
47	51.9	chondrillasterol	87	3300	3295
48	52.1	β-amyrone	89	3313	3327
49	52.5	β-amyrin	93	3340	3337
50	52.8	α-amyrone	89	3360	3373
51	54.7	pseudotaraxasterol	89	3476	-

ID, peak identification; Rt, retention time in minutes; S, similarity index; RI_{calc}, calculated retention index; RI_{ref}, retention index found in the NIST website (https://webbook.nist.gov/chemistry/name-ser/).

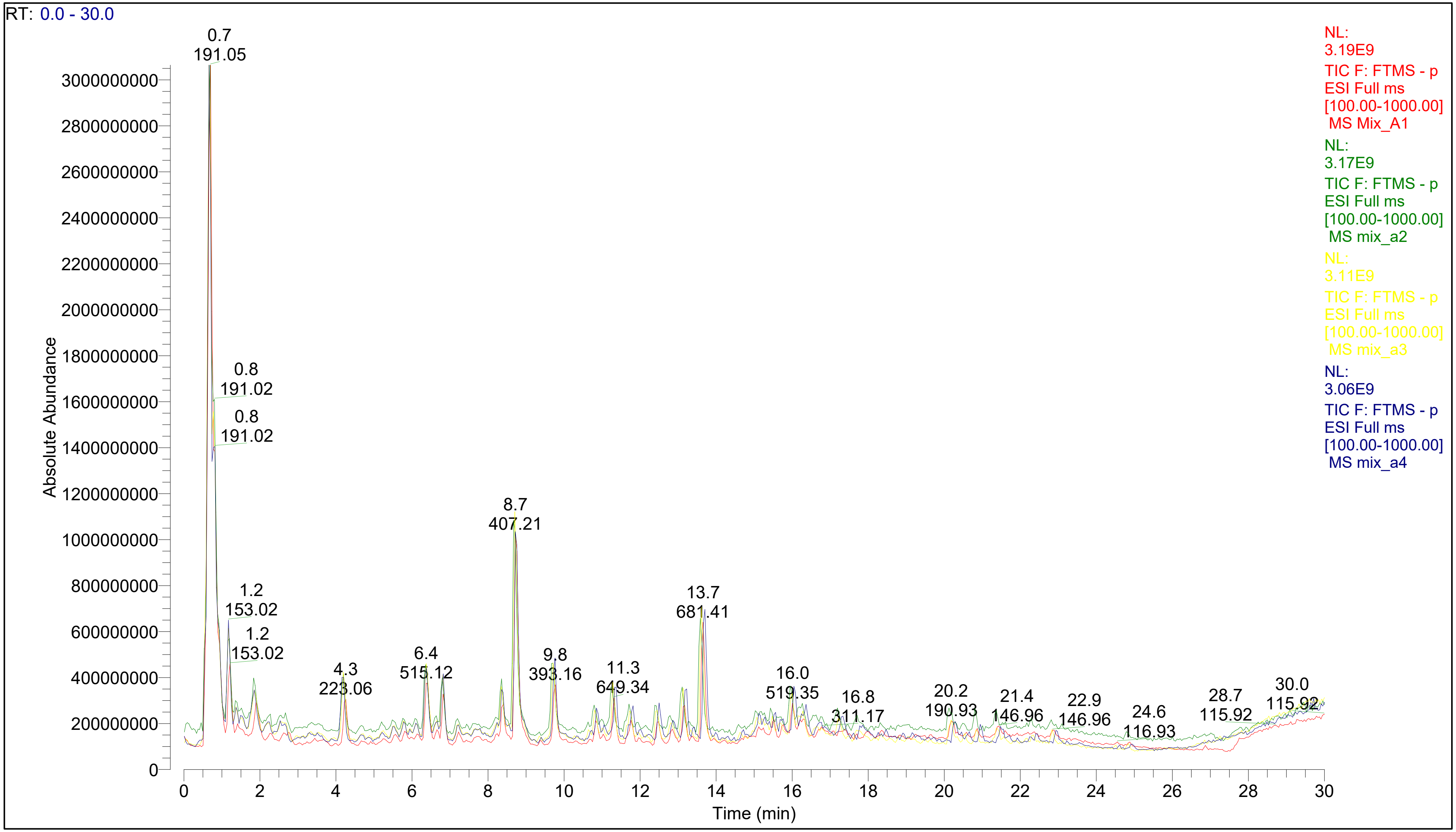

Figure S1: Total ion chromatograms (TIC) of the quality control samples analyzed by UHPLC-UV-MS during the data acquisition in the negative ionization mode.

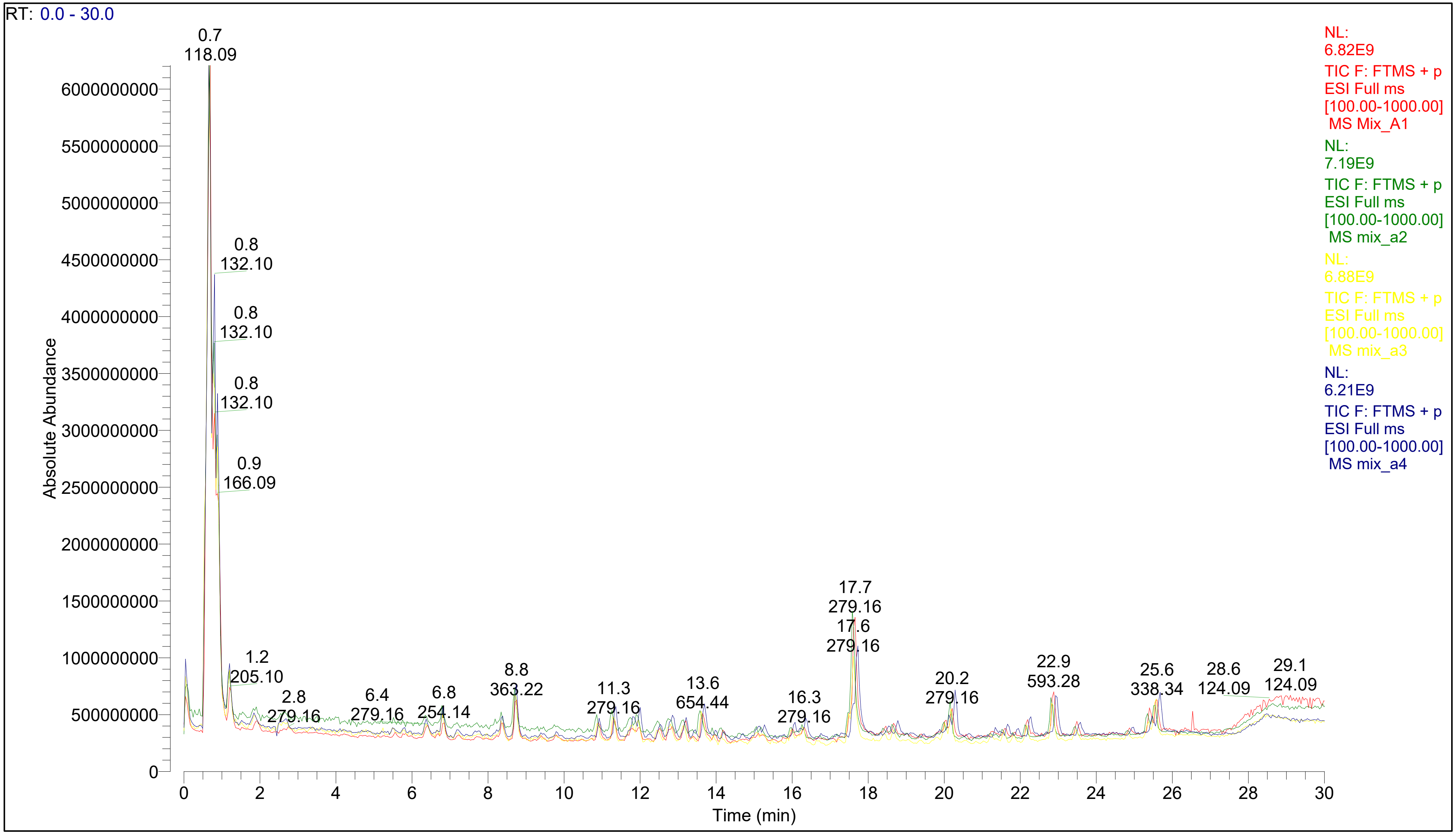

Figure S2: Total ion chromatograms (TIC) of the quality control samples analyzed by UHPLC-UV-MS during the data acquisition in the positive ionization mode.
